# Cardiovascular safety of lumiracoxib: a meta-analysis of randomised controlled trials in patients with osteoarthritis

**DOI:** 10.1007/s00228-012-1335-1

**Published:** 2012-06-26

**Authors:** Isla S. Mackenzie, Li Wei, Thomas M. MacDonald

**Affiliations:** Medicines Monitoring Unit (MEMO) & Hypertension Research Centre (HRC), Division of Medical Sciences, University of Dundee, Ninewells Hospital & Medical School, Dundee, DD1 9SY UK

**Keywords:** Lumiracoxib, Cyclooxygenase, NSAIDs, Cardiovascular, Osteoarthritis, Hypertension

## Abstract

**Purpose:**

To re-evaluate the cardiovascular risk of lumiracoxib compared with other non-steroidal anti-inflammatory drugs (NSAIDs) or placebo in patients with osteoarthritis.

**Methods:**

We conducted a meta-analysis of randomised controlled trials of lumiracoxib versus placebo or other NSAIDs in patients with osteoarthritis reported up to January 2010. Both published and unpublished trials were included. PubMed searches using predefined search criteria (lumiracoxib AND osteoarthritis, limits: none; COX-189 AND osteoarthritis, limits: none) were used to obtain the relevant published trials. Novartis granted explicit access to their company studies and the right to use these study reports for the purposes of publication in peer reviewed journals. Endpoints were the Antiplatelet Trialists’ Collaboration (APTC) endpoint and individual cardiovascular endpoints.

**Results:**

Meta-analysis of 6 trials of lumiracoxib versus placebo revealed no difference in cardiovascular outcomes. Meta-analysis of 12 trials of lumiracoxib versus other NSAIDs also revealed no difference. The pooled odds ratios were: 1.16 (95% CI 0.82, 1.63); 1.66 (95% CI 0.84, 3.29); 0.95 (95% CI 0.52, 1.76) and 1.04 (95% CI 0.60, 1.80) for the APTC endpoint, myocardial infarction, stroke and cardiovascular death respectively.

**Conclusions:**

The results suggest that there were no significant differences in cardiovascular outcomes between lumiracoxib and placebo or between lumiracoxib and other NSAIDs in patients with osteoarthritis. Wide confidence intervals mean that further research is needed in this area to confirm these findings.

## Introduction

Lumiracoxib (COX-189) is a cyclooxygenase type 2 (COX-2) inhibitor and non-steroidal anti-inflammatory drug (NSAID) used for the treatment of osteoarthritis and acute pain. As a group, NSAIDs, including both traditional non-selective NSAIDs and COX-2 selective inhibitors, have been associated with increased risk of cardiovascular events [1]. Possible mechanisms have been debated widely and probably include effects on platelet function, blood pressure and sodium retention.

Different NSAIDs have different effects on specific cardiovascular parameters such as blood pressure. Lumiracoxib may have certain advantages over other NSAIDs in terms of its effects on blood pressure [2, 3]. However, lumiracoxib has been associated with severe liver injury in a small number of patients, some of whom required liver transplantation. In many of these cases other risk factors for liver disease were also present and it was not clear whether the liver injury was drug-induced. The result was that lumiracoxib was withdrawn from several worldwide markets from 2007 onwards and it also failed to gain approval in other countries because of the potential for hepatotoxicity. Recently, a genome-wide association (GWA) study identified HLA alleles strongly associated with risk of hepatotoxicity with lumiracoxib, opening up the possibility of pre-treatment pharmacogenetic screening to exclude patients at higher risk of liver injury from lumiracoxib treatment [4].

This paper re-evaluates the cardiovascular safety of lumiracoxib in patients with osteoarthritis in comparison to other NSAIDs and placebo at a time when it looks possible that lumiracoxib might re-emerge on to the market alongside a pharmacogenomic screening test to target its use more safely.

## Materials and methods

A systematic review of clinical trials of lumiracoxib in patients with osteoarthritis reported up to January 2010 was undertaken. Trials were included if they used lumiracoxib daily doses of 100–400 mg, were of at least 1 week’s duration and if they had a substantial cardiovascular component. Both published and unpublished trials were included. Novartis granted explicit access to their company studies and the right to use these study reports for the purposes of publication in peer reviewed journals. PubMed searches using predefined search criteria (lumiracoxib AND osteoarthritis, limits: none; COX-189 AND osteoarthritis, limits: none) were used to obtain the relevant published trials. Where both were available, published papers were matched to the relevant company clinical study reports to avoid double inclusion of the same study data. Each study was graded according to the quality of evidence using an appropriate validated grading system, the Jadad scale [5]. Only studies judged to be of sufficient quality (Jadad score >3) were included in the evaluation. The list of published trials and clinical study reports with Jadad scores >3 that were included for detailed review and meta-analysis in this report is presented in Table 1. Cardiovascular events were the primary outcome for only one study [6] and were reported as adverse events in others. Adverse events were reviewed by an independent safety committee in a blinded manner for 4 studies [2, 6–8]. In the other studies AEs were reported as recorded by investigators or subjects. All patients had to meet the inclusion criterion of primary osteoarthritis and patients who had secondary osteoarthritis, other connective tissue diseases, or significant medical problems were excluded from the studies. The average age of the study participants in Table 1 ranged from 59.5 to 65.5 years old and there were more women than men in the studies (ranging from 59% to 76% across the studies). Safety assessment was carried out for the duration of the studies. One study reported a safety assessment lasting 2 weeks after the end of study. The studies that were considered but excluded are listed in Table 2, along with the Jadad scores assigned and the reasons for exclusion. The initial search of studies and Jadad score assignment were carried out by Chameleon Communications International under our instruction. We subsequently independently reviewed and approved this work.

**Table 1:** List of studies included for review

Reference (published studies)	Study number (Novartis clinical study reports)	Total number of patients	Lumiracoxib dose (number of patients)	Comparator NSAIDs and dose (number of patients)	Exposure duration (days)	Jadad score
[9]	CSR104	583	50 mg bd (98)	Placebo (97)	28	4^a^/5
			100 mg bd (96)	Diclofenac 75 mg bd (94)		
			200 mg bd (99)			
			400 mg od (99)			
–	CSR109	1,600	200 mg od (462)	Placebo (231)	91	5
			400 mg od (463)	Celecoxib 200 mg od (444)		
[10]	CSR112	1,702	200 mg od (487)	Placebo (243)	91	4^a^/5
			400 mg od (491)	Celecoxib 200 mg od (481)		
–	CSR112E	1,235	200 mg od (411)	Celecoxib 200 mg od (405)	273	5
			400 mg od (419)			
–	CSR126	1,042	200 mg od (264)	Ibuprofen 800 mg tds (260)	91	5
			400 mg od (260)	Celecoxib 200 mg od (258)		
–	CSR128	511	400 mg od (205)	Placebo (204)	91	5
				Rofecoxib 25 mg od (102)		
–	CSR2301	364	400 mg od (144)	Placebo (75)	7	5
				Celecoxib 200 mg bd (145)		
–	CSR2303	408	200 mg od (105)	Placebo (103)	7	5
			400 mg od (99)	Celecoxib 200 mg bd (101)		
[11]	CSR2307	309	400 mg od (154)	Rofecoxib 25 mg od (155)	42	5^a^/5
–	CSR2316	244	100 mg od (122)	Placebo (122)	28	5
–	CSR2319	594	200 mg od (205)	Placebo (196)	28	5
			400 mg od (193)			
[7]	CSR2360	1,551	100 mg od (391)	Placebo (382)	91	5^a^/5
			200 mg od (385)	Celecoxib 200 mg od (393)		
[8]	CSR2361	1,684	100 mg od (420)	Placebo (424)	91	5^a^/5
			200 mg od (420)	Celecoxib 200 mg od (420)		
–	CSR2361E	1,310	100 mg od (853)	Celecoxib 200 mg od (457)	273	5
–	CSR2364	703	200 mg od (352)	Celecoxib 200 mg od (351)	42	5
–	CSR2367	1,262	100 mg od (427)	Placebo (416)	91	5
				Celecoxib 200 mg od (419)		
–	CSR2369	3,036	100 mg od (757)	Celecoxib 200 mg od (759)	364	5
			100 mg bd (1,520)			
[2]	CSR2428	787	100 mg od (394)	Ibuprofen 600 mg tds (393)	28	5^a^/5
[6]	TARGET 0117 and 2332	18,325	400 mg od (9,156)	Ibuprofen 800 mg tds (4,415)	365	5^a^/5
				Naproxen 500 mg bd (4,754)		

bd = twice daily; NSAID = non-steroidal anti-inflammatory drug; od = once daily; tds = three times daily

^a^Jadad score assigned to published paper

**Table 2:** List of all studies considered and excluded with Jadad scores and reasons for exclusion

Reference	Jadad score	Reason for exclusion (if applicable)
Published studies		
Fleischmann, R et al. BMC Musculoskelet Disord 2008;9:32	1	Jadad score ≤3
Bin, Seong-II et al. APLAR J Rheumatol 2007;10:190	3	Jadad score ≤3
Farkouh, ME et al. J Clin Hypertens (Greenwich) 2008;10:592	Not applicable	Post-hoc analysis of data in Farkouh et al .2004
Farkouh, ME et al. Ann Rheum Dis 2007;66:764	Not applicable	Extended report of data in Farkouh et al. 2004
Kyle, C et al. Int J Clin Pract 2008;62:1684	4	No significant CV component
Sheldon, EA et al. Clin Exp Rheumatol 2008;26:611	2	No significant CV component
Wittenberg, RH et al. Arthritis Res Ther 2006;8:R35	3	No significant CV component
Dougados, M et al. Arthritis Res Ther 2006;9:R11	5	No significant CV component
Fleischmann, R et al. Clin Rheumatol 2006;25:42	3	No significant CV component
Grifka, JK et al. Clin Exp Rheumatol 2004;22:589	3	No significant CV component
Hawkey, CJ et al. Aliment Pharmacol Ther 2008;27:838	5	No significant CV component
Hawkey, CJ et al. Gastroenterology 2007;133:57	Not applicable	No significant CV component
Schnitzer, TJ et al. Lancet 2004;364:665	5	No significant CV component
Schnitzer, TJ et al. Curr Med Res Opin 2005;21:151	2	Combined analysis
Matchaba, P et al. Clin Ther 2005;27:1196	Not applicable	Meta-analysis
Chen, YF et al. Health Technol Assess 2008;12:1	0	Study in patients with RA
Hawkey, CJ et al. Clin Gastroenterol Hepatol 2006;4:57	Not applicable	Study in patients with RA
Nielsen, OH et al. Aliment Pharmacol Ther 2006;23:27	0	Study in patients with RA
Berenbaum, F et al. J Int Med Res 2005;33:21	3	In vitro study
Geusens, P et al. Int J Clin Pract 2004;58:1033	3	Study in patients with RA
Kivitz, AJ et al. Aliment Pharmacol Ther 2004;19:1189	3	Study in patients with RA
Scott, G et al. Clin Pharmacokinet 2004;43:467	0	Study in patients with RA
Kang, P et al. Chem Res Toxicol 2009;22:106	0	In vitro study
Laine, L et al. Semin Arthritis Rheum 2008;38:165	0	Review article
Shi, S et al. Eur J Clin Pharmacol 2008;64:233	0	Review article
Aust Nurs J 2007;15:8 [no authors listed]	0	Commentary
Burton, B et al. BMJ 2007;335:363	0	Commentary
Baraf, HS et al. Curr Pharm Des 2007;13:2228	0	Review article
Bannwarth, B et al. Expert Opin Pharmacother 2007;8:1551	0	Review article
Hochberg, MC et al. Curr Top Med Chem 2005;5:443	0	Review article
Bannwarth, B et al. Expert Opin Investig Drugs 2005;14:521	0	Review article
Z Orthop Ihre Grenzgeb 2005;143:158 [no authors listed]	Not applicable	Article not in English
Rordorf, CM et al. Clin Pharmacokinet 2005;44:1247	0	Review article
Rosenberg, JA et al. Nat Clin Pract Gasteroenterol Hepatol 2005;2:14	0	Review article
Hart, L et al. ACP J Club 2005;142:46	0	Commentary
Health News 2004;10:13 [no authors listed]	0	Commentary
Kiefer, W et al. Curr Med Chem 2004;11:3147	0	Review article
Summerton, N et al. Br J Gen Pract 2004;54:880	0	Review article
Lyseng-Williamson, KA et al. Drugs 2004;64:2237	0	Review article
Hawkey, CC et al. J Rheumatol 2004;31:1804	0	Review article
Lazzaroni, M et al. Aliment Pharmacol Ther 2004;20:48	0	Review article
Topol, EJ et al. Lancet 2004;364:639	0	Commentary
Mysler, E et al. Int J Clin Pract 2004;58:606	0	Review article
Hawkey, CJ et al. Aliment Pharmacol Ther 2004;20:51	0	Trial design only
Mangold, JB et al. Drug Metab Dispos 2004;32:566	0	Study in healthy volunteers
Tacconelli, S et al. Curr Pharm Res 2004;10:589	0	Review article
Capone, ML et al. Int J Immunopathol Phramacol 2003;16:49	0	Review article
Ding, C et al. IDrugs 2002;5:1168	0	Review article
Buvanendran, A et al. Drugs Today (Barc) 2007;43:137	0	Review Article
Ker, J et al. Cardiovasc J Afr 2007;18:383	0	Case study
EULAR abstracts		
Fleischmann, R et al. EULAR 2003 Poster FRI0233	0	Not full publication
Schell, E et al. EULAR 2003 Poster FRI0224	2	Not full publication
Benevolenskaya, L et al. EULAR 2003 Poster FRI0246	0	Not full publication
Grifka, JK et al. EULAR 2003 Poster FRI0222	0	Not full publication
Pavelka, K. EULAR 2005 Poster FRI0319	0	Not full publication
Clinical study reports		
CSR105	5	Study in patients with RA
CSR110	5	Study in patients with RA
CSR111	5	Study in patients with RA
CSR 114	5	Study in patients with RA
CSR 2312	5	Study in patients with RA
CSR 2335	5	Study in patients with RA
CSR 2360E	1	No comparator
CSR 2365	1	No comparator
CSR 2425	5	Study in healthy subjects
CSR 2427	5	Study in patients undergoing knee surgery

RA = rheumatoid arthritis; CV = cardiovascular; EULAR = European League Against Rheumatism; CSR = Clinical Study Report

Cardiovascular endpoints of relevance to the present paper primarily included the Antiplatelet Trialists’ Collaboration (APTC) events (non-fatal myocardial infarction [MI], non-fatal stroke or cardiovascular death). However, each of the APTC components was also considered separately where data were available. Tabulations of the events of interest and crude event rates were abstracted. Subject level data were generally not available, although some descriptions of individual serious adverse events and deaths were provided in clinical study reports. Overall crude summary statistics were constructed.

### Data extraction

Data extraction from the published studies and clinical study reports was performed by one author and checked by the other authors. Although in many cases, there was clear reporting of numbers of events, in a few cases, a value judgement had to be applied as to whether to include events or not, for example, when events occurred soon after completion of the study.

### Statistical analysis

The odds ratio (OR) and 95% confidence intervals (CIs) were calculated for each trial based on the total number of patients and total number of events in each group. The fixed effects model was used to obtain pooled ORs after a heterogeneity test among the trials. Publication bias was assessed by Egger’s test and Begg’s funnel plots for each of the endpoints studied by meta-analysis and no bias was found. The meta-analysis was conducted using StatsDirect software.

## Results

### Characteristics of the studies included in the review

All the studies included were randomised controlled trials in patients with osteoarthritis. All studies were of lumiracoxib versus placebo or lumiracoxib versus one or more active NSAID comparators (or both placebo and NSAID comparator). Doses of lumiracoxib were between 50 mg twice daily and 400 mg once daily (total daily doses 100–400 mg). Comparator NSAIDs in the studies included ibuprofen, celecoxib, naproxen, rofecoxib and diclofenac. The duration of drug therapy in the studies was from 1 week to 1 year. All the studies included were of sufficient quality to be graded with a Jadad score of >3. Of the 19 studies included, 7 were reported as both published journal articles and clinical study reports [2, 6–9, 10–12] and the remainder were reported only in unpublished Novartis clinical study reports. Where both the published journal article and the original clinical study reports were available pertaining to the same data, both reports were reviewed, but the data were only included once.

### Randomised controlled trials of lumiracoxib versus placebo

Of the studies included, 11 out of 19 compared lumiracoxib with placebo; only 6 of these reported the occurrence of any APTC events and event numbers were very small, providing limited data for further analysis. Other cardiovascular endpoints were not reported consistently among studies and again, numbers of events were very small. Therefore, meta-analysis was limited to the APTC endpoints for this group of trials and was not conducted for any other individual cardiovascular outcomes.

The 6 studies comparing lumiracoxib versus placebo and reporting any APTC events are listed in Table 3. Only 6 APTC events were recorded in 4,122 lumiracoxib users and 1 APTC event in 1,680 placebo users. In total, there were 959 person-years’ exposure to lumiracoxib and 385 person-years’ exposure to placebo within this group of studies. The meta-analysis from the 6 trials revealed no significant difference in the incidence of APTC endpoints between lumiracoxib and placebo (pooled OR 1.10, 95% CI 0.31, 3.94). However, the wide confidence intervals did not exclude lumiracoxib being almost 70% better than placebo or 394% worse than placebo.

**Table 3:** Antiplatelet Trialists’ Collaboration (APTC) events in the lumiracoxib and placebo groups

Novartis study number	Reference (if published)	Number of patients in the lumiracoxib group	Number of patients in the placebo group	Number of APTC events in the lumiracoxib group	Number of APTC events in the placebo group
CSR109	–	925	231	1	0
CSR128	–	205	204	1	1
CSR2319	–	398	196	1	0
CSR2361	[8]	840	424	1	0
CSR2360	[7]	776	382	1	0
CSR112	[10]	978	243	1	0
Total		4,122	1,680	6	1

### Randomised controlled trials of lumiracoxib versus other NSAIDs

There were 17 trials comparing lumiracoxib with other NSAID comparators. Of these, 12 studies reported any occurrence of APTC events in either group. There were sufficient data to perform meta-analysis for APTC events, MI, stroke and cardiovascular death. In total, there were 13,256 person-years’ exposure to lumiracoxib and 10,964 person-years’ exposure to other NSAID comparators within this group of studies.

**Fig. 1:**
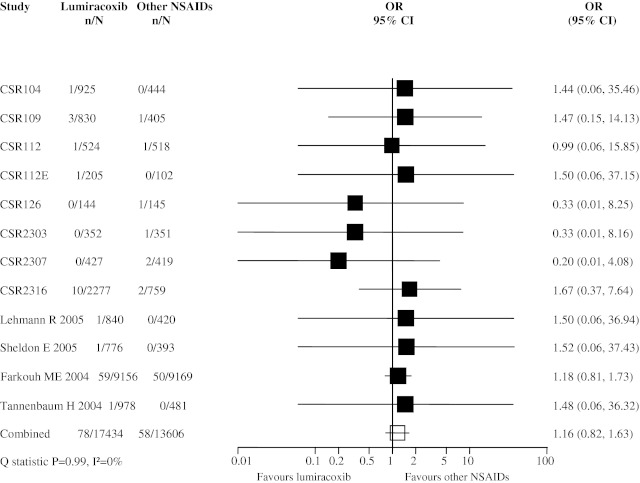
Meta-analysis of APTC endpoints in randomized controlled trials comparing lumiracoxib with other non-steroidal anti-inflammatory drugs (NSAIDs)

There were 12 trials reporting any occurrence of APTC endpoints. In total, 78 events were reported in the lumiracoxib group (*n* = 17,434) and 58 events in the active NSAID comparator groups (*n* = 13,606). The pooled OR for the likelihood of APTC endpoints with lumiracoxib versus other NSAIDs was 1.16 (95% CI 0.82, 1.63; Fig. 1). A sensitivity analysis, excluding 8 trials in which there were no events in either one of the lumiracoxib or comparator NSAIDs arms, showed a similar result (pooled OR 1.21, 95% CI 0.84, 1.73).

**Fig. 2:**
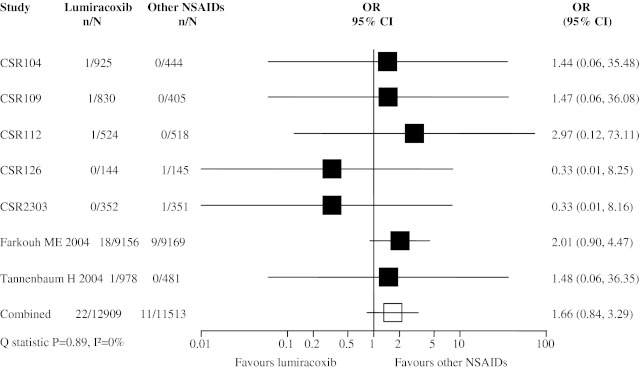
Meta-analysis of myocardial infarction (MI) events in randomized controlled trials comparing lumiracoxib with other NSAIDs

Data for the MI endpoint were available in 7 trials with a total of 33 MI events reported in 24,422 patients (12,909 in the lumiracoxib group and 11,513 in the other NSAIDs group). Overall, there was no significantly increased risk of MI for lumiracoxib users (pooled OR 1.66, 95% CI 0.84, 3.29; Fig. 2). Six trials had only one MI event in either the lumiracoxib group or the other NSAID group. Farkouh et al. was the driving study for the pooled result.

**Fig. 3:**
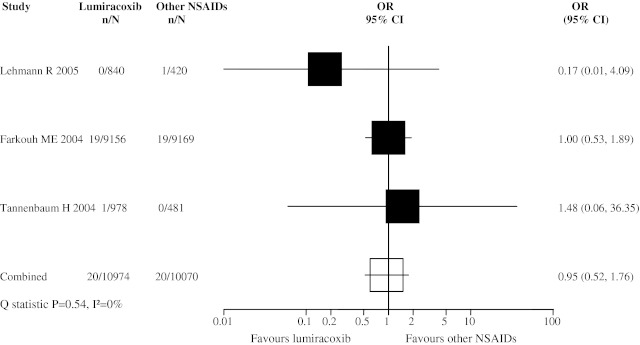
Meta-analysis of stroke events in randomized controlled trials comparing lumiracoxib with other NSAIDs

Stroke occurrence was rare within the trials studied. Only 3 trials were included in the meta-analysis (Fig. 3). The pooled OR for stroke was 0.95 (95% CI 0.50, 1.76).

**Fig. 4:**
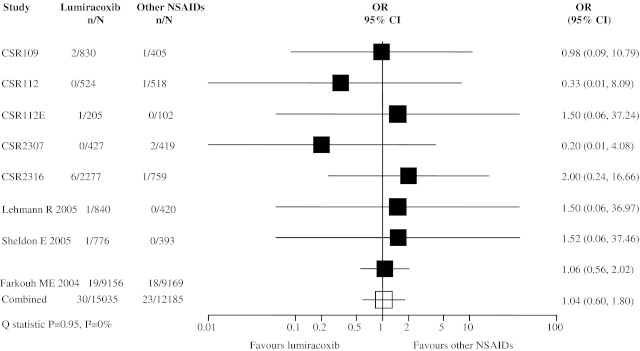
Meta-analysis of cardiovascular death events in randomized controlled trials comparing lumiracoxib with other NSAIDs

Eight trials reported cardiovascular death events and meta-analysis found no increased risk of cardiovascular death in the lumiracoxib group compared with the other NSAID group (Fig. 4; OR 1.04, 95% CI 0.60, 1.80). A sensitivity analysis excluding 5 trials with no events occurring in either group resulted in a slightly higher estimate of the pooled OR (OR 1.11, 95% CI 0.61, 2.01), but this remained statistically non-significant.

## Discussion

Having reviewed the published and unpublished data available, it is apparent that while several clinical trials have been performed, we still have a paucity of data to judge the cardiovascular safety of lumiracoxib versus placebo or other NSAIDs, because cardiovascular events were rare in most of the studies. As far as we are able to judge from the data available, there is no evidence of increased risk of cardiovascular events with lumiracoxib versus placebo or versus other NSAIDs. However, for example for the MI endpoint, because of the wide confidence intervals, the results are compatible with a 16% lower or 229% higher risk of MI with lumiracoxib versus other NSAIDs.

Our results are in agreement with a previous meta-analysis by Matchaba et al., which examined the cardiovascular safety of lumiracoxib in randomised controlled trials in patients with osteoarthritis or rheumatoid arthritis [13]. It found no evidence that lumiracoxib was associated with a significant increase in cardiovascular risk compared with naproxen, placebo or all comparators (placebo, diclofenac, ibuprofen, celecoxib, rofecoxib and naproxen).

Non-steroidal anti-inflammatory drugs increase cardiovascular risk to differing extents. Although the studies we used in our analysis included several different NSAID comparators, the total number of events occurring for each individual NSAID was small. Therefore, we were not able to draw conclusions about the relative safety of individual NSAID comparators and lumiracoxib, but rather grouped the comparator NSAIDs together for our analysis. Similarly, there was insufficient event data to allow analysis of dose effects within either the lumiracoxib or NSAID comparator groups. While in several studies there is a trend towards some NSAIDs being safer, e.g. naproxen, and some being more harmful, e.g. rofecoxib, the mechanisms of increased risk are likely to be mixed and to include vascular, platelet and blood pressure effects. In patients with osteoarthritis, a risk–benefit judgement must be made to balance pain control and quality of life with any potential side effects of NSAIDs.

At least some of the blood pressure effects of NSAIDs are likely to be due to sodium retention [14]. Interestingly, while modest dietary salt restriction leads to significant reductions in systolic and diastolic blood pressures (average of up to 5 and 3 mmHg respectively) in patients with hypertension [15, 16], a low salt diet leads to a much more striking reduction in blood pressure in patients with resistant hypertension (average 22.7 and 9.1 mmHg reductions in office systolic and diastolic pressures respectively) [17]. Similarly, the potential benefits of lumiracoxib compared with other NSAIDs on blood pressure could be greatest in those patients with resistant hypertension. Therefore, lumiracoxib may be most beneficial for patients with osteoarthritis requiring NSAID therapy for pain control, but who also have resistant hypertension.

Limitations of our meta-analysis include the small number of cardiovascular events occurring in most of the trials. From the data, we calculated that the estimated incidences of APTC events for the active comparator group and the lumiracoxib group were 5.29 and 5.88 per 1,000 person-years respectively. It would require 29,486 person-years’ exposure and 195 events to detect a 50% difference in the incidence of APTC events at a 5% significance level and 80% power between lumiracoxib and other NSAIDs. Clearly our meta-analysis is not powered to detect such a difference. By including only higher quality trials (Jadad score >3) we may have excluded some other data, although one could argue that lower quality trials should not be included anyway. Our meta-analysis was limited to patients with osteoarthritis, who are generally thought to be at lower cardiovascular risk than patients with rheumatoid arthritis. A recent network meta-analysis including trials in patients taking NSAIDs for any medical condition found a statistically significant increase with lumiracoxib versus placebo in the rate ratio of stroke RR 2.81 (95% CI 1.05–7.48) and of the APTC endpoint RR 2.04 (1.13–4.24), but no increased risk of MI, cardiovascular death or death of any cause [12]. Using the network meta-analysis technique to allow indirect comparisons between different NSAIDs, no statistically significant increase in risk of any of the above endpoints was found with lumiracoxib versus naproxen, etoricoxib, celecoxib, rofecoxib, diclofenac or ibuprofen.

## Conclusions

The results suggest that the cardiovascular risk with lumiracoxib was not significantly different from that with placebo or with other NSAIDs. Wide confidence intervals mean that further research is needed in this area to confirm these findings.
